# High-Resolution Denitrification Kinetics in Pasture Soils Link N_2_O Emissions to pH, and Denitrification to C Mineralization

**DOI:** 10.1371/journal.pone.0151713

**Published:** 2016-03-18

**Authors:** Md Sainur Samad, Lars R. Bakken, Shahid Nadeem, Timothy J. Clough, Cecile A. M. de Klein, Karl G. Richards, Gary J. Lanigan, Sergio E. Morales

**Affiliations:** 1 Department of Microbiology and Immunology, Otago School of Medical Sciences, University of Otago, Dunedin, New Zealand; 2 Department of Environmental Sciences, Norwegian University of Life Sciences, Ås, Norway; 3 Department of Soil and Physical Sciences, Lincoln University, Lincoln, New Zealand; 4 AgResearch Invermay, Mosgiel, New Zealand; 5 Teagasc, Environmental Research Centre, Johnstown Castle, Wexford, Ireland; USDA-ARS, UNITED STATES

## Abstract

Denitrification in pasture soils is mediated by microbial and physicochemical processes leading to nitrogen loss through the emission of N_2_O and N_2_. It is known that N_2_O reduction to N_2_ is impaired by low soil pH yet controversy remains as inconsistent use of soil pH measurement methods by researchers, and differences in analytical methods between studies, undermine direct comparison of results. In addition, the link between denitrification and N_2_O emissions in response to carbon (C) mineralization and pH in different pasture soils is still not well described. We hypothesized that potential denitrification rate and aerobic respiration rate would be positively associated with soils. This relationship was predicted to be more robust when a high resolution analysis is performed as opposed to a single time point comparison. We tested this by characterizing 13 different temperate pasture soils from northern and southern hemispheres sites (Ireland and New Zealand) using a fully automated-high-resolution GC detection system that allowed us to detect a wide range of gas emissions simultaneously. We also compared the impact of using different extractants for determining pH on our conclusions. In all pH measurements, soil pH was strongly and negatively associated with both N_2_O production index (*I*N_2_O) and N_2_O/(N_2_O+N_2_) product ratio. Furthermore, emission kinetics across all soils revealed that the denitrification rates under anoxic conditions (NO+N_2_O+N_2_ μmol N/h/vial) were significantly associated with C mineralization (CO_2_ μmol/h/vial) measured both under oxic (*r*^2^ = 0.62, *p* = 0.0015) and anoxic (*r*^2^ = 0.89, *p*<0.0001) conditions.

## Introduction

Nitrous oxide (N_2_O) is a potent greenhouse gas contributing 8% of anthropogenic global warming [1,2,3] and responsible for depleting stratospheric ozone [4]. The N_2_O molecule has a Global Warming Potential (GWP) 298 times higher than carbon dioxide (CO_2_) over a 100-year period and an atmospheric life of approximately 121 years [3]. In the atmosphere, N_2_O has increased by 20% over the last 260 years (1750 to 2011) from 271 ppb to 324 ppb [5]. Currently, the major anthropogenic source of N_2_O is agricultural soils [6,7]. In these N_2_O emitting soils denitrification is thought to be the most important pathway leading to N_2_O loss [8,9], although a recent study showed that ammonia oxidation pathways and nitrifier denitrification are significant sources of N_2_O and NO under low oxygen availability [10].

Denitrification is the stepwise process of reducing nitrate (NO_3_^-^) to N_2_O or N_2_, via nitrite (NO_2_^-^) and nitric oxide (NO). Four reductase enzymes catalyse the steps: nitrate reductase (NAR), nitrite reductase (NIR), nitric oxide reductase (NOR) and nitrous oxide reductase (N_2_OR) [11,12]. The key requirements for biological denitrification, and complete reduction of nitrate to N_2_, can be summarized into two components: 1) the presence of microbes harbouring the genetic ability to perform all the steps in denitrification, and 2) suitable environmental conditions for expression of the genetic potential. Changes in these two components can modify N_2_O emissions from soils [13,14]. For example, some organisms (complete denitrifiers) contain all the genetic information needed to produce the four enzymes, while others (incomplete denitrification) lack a subset of the enzymes and can only catalyse portions of the denitrification process [11,12]. Alternatively, changes in the concentration and ratio of electron donors (i.e. available organic carbon compounds), available terminal electron acceptors (e.g. NO_3_^-^, NO_2_^-^, NO or N_2_O), and soil redox potential can modulate environmental conditions and thus the efficiency of denitrification in soils [13,15]. The addition of nitrogen fertilizers or manures increases denitrification rates especially when there is an adequate supply of carbon [16,17]. This is due to the fact that, denitrifiers require C to be readily available for reduction of NO_3_^-^ to occur [17]. The rate of C mineralisation in soils is influenced by many factors (e.g. temperature, drying-wetting, tillage, liming, crop residues, fertiliser application, root exudates) and which ultimately have a major impact on the denitrification rate [13].

Known regulators can be difficult to assess in agricultural settings, and even more complicated to manipulate. An important factor that is more amenable for manipulation, and is a strong regulator of soil denitrification at both proximal and distal scales, is pH [18]. Soil pH is a key driver of the microbiological processes affecting N_2_O and N_2_ production [12,13], and influences the N_2_O/(N_2_O+N_2_) product ratio and N_2_O production index of soils. Proximal control by pH implicates direct changes in N_2_O-reductase activity, while distal control by pH implicates changes in the denitrifier community, which is an important component affecting N_2_O emission rates [18]. The mechanisms producing such effects are not well understood however, recent findings based on gene transcription, protein expression and the kinetics of electron flow at the cellular level have provided promising clues. In the model organism *Paracoccus denitrificans* environmental pH hinders the posttranslational assembly of a functional N_2_O-reductase enzyme [12,19,20]. The inactivity of this enzyme results in the accumulation of N_2_O, which in results in soils becoming net N_2_O sources. Since soil pH can be controlled at field scales it represents a potential tool for mitigating N_2_O emissions from soils, but integrating knowledge across studies is made complicated due to variations in methodologies, most commonly the type of extractant used for pH measurements. Several different extractants (e.g. water, CaCl_2_ and KCl) are widely used for measuring soil pH [21,22]. However, the KCl based pH measurement is less commonly used for agricultural soils because its strong nature can alter the original properties of the sample being studied [22]. This variability limits our capacity to integrate results over studies since the effects these changes can have on measurements are not fully understood.

Here we used a fully automated high-resolution GC detection system for measuring gas emissions under standardized oxic and anoxic conditions in order to assess factors linked to pasture soil N_2_O emission and denitrification potential across soils representing both Northern and Southern hemispheres. Our objectives were: (1) to determine the denitrification kinetics of pasture soils, (2) to determine the effect changing methods (extractant type) for determining soil pH has on observed relationship with N_2_O flux, (3) to compare two methods of quantifying N_2_O emissions from soils (an emission index and ratio), and (4) to investigate the relationship between denitrification and C mineralization in soils.

## Materials and Methods

**Fig 1:**
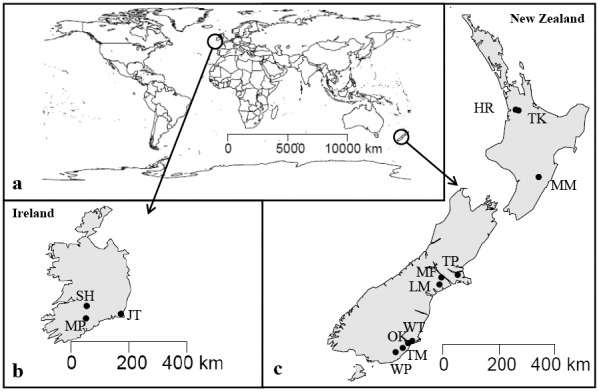
Geographical location of soil samples. Map showing origin of soil samples used in the study (a) world map, (b) Ireland [Moorepark (MP), Johnstown (JT), Solohead (SH)] and (c) New Zealand [Warepa (WP), Otokia (OT), Wingatui (WT), Tokomairiro (TM), Mayfield (MF), Lismore (LM), Templeton (TP), Manawatu (MM), Horotiu (HR), Te Kowhai (TK)]. The map was generated using open source “R-programme (packages ‘maps’ and ‘mapdata’)”.

Soil samples were collected from 13 different sites (Fig 1) in the Northern and Southern hemispheres: (Ireland- Moorepark, Johnstown, Solohead and New Zealand- Warepa, Otokia, Wingatui, Tokomairiro, Mayfield, Lismore, Templeton, Manawatu, Horotiu, Te Kowhai). Soil properties are presented in S1 Table. Permission for sampling was not required or in the case of sites located on private land, owner permission was secured for sampling.

At each site multiple (>3) soil cores (25 mm diameter by 100 mm long, and excluding the grass layer) were collected and sieved to 2–4 mm, composited and immediately couriered to the Norwegian University of Life Sciences, Norway for analysis. Soil samples were stored at 4°C in the lab until analysed (within one week).

### Soil pH measurements

Soil pH was measured using three different extraction methods: i) deionised (DI) water, ii) 0.01 M CaCl_2_ and iii) 2M KCl. All pH measurements were carrying out using a 10 ml soil sample (field moist) measured using a volumetric spoon and transferred to a plastic vial. The respective pH treatment solutions (DI water, 0.01 M CaCl_2_ or 2M KCl) were added (25 ml) and the vials were sealed and then, mixed thoroughly by hand shaking for 1 minute and left to settle overnight. Immediately prior to measuring the pH, samples were shaken well and allowed to settle for 10 minutes. All pH measurements were done using an Orion 2-star pH Benchtop pH meter (Thermo Scientific) equipped with an Orion 8175BNWP electrode (Thermo Scientific).

### Nitrate adjustment

Individual soil samples (100 g dry weight) were placed in 500 ml filter funnels (Millipore) with 4.5 cm diameter (0.2 μm) Millipore membrane filters and subsequently flooded with a 2 mM NH_4_NO_3_ solution for 10 minutes. Samples were then drained using a vacuum in order to obtain a homogeneous distribution of NO_3_^-^ in the soils. The moisture content of the soil samples was determined after draining and dry weight equivalents were used for subsequent gas kinetic experiments.

### Gas kinetics under oxic and anoxic conditions

All incubations were performed using slightly modified methods described previously [23–25]. In brief, following NO_3_^-^ adjustment, 20 g (dry weight equivalent) of soil was transferred to a 120 ml serum vial and sealed with an airtight butyl-rubber septa and an aluminium crimp cap. Triplicate vials were prepared from each soil sample and incubated at 20°C using an automated GC system [25]. The GC (Agilent GC -7890A) system was equipped with three detectors (an electron capture detector (ECD), a thermal conductivity detector (TCD), a flame ionization detector (FID)) and one Chemiluminescence NOx analyser (NOx analyser Model 200A, Advanced Pollution Instrumentation, San Diego, USA). The GC system was integrated with an automated sampling robot (CTC GC PAL). All data presented were from experiments performed over two runs, which included independent standards for each run. Duplicates of four different gas standards were used in this experiment. All standards were prepared using evacuated vials (120 ml with septum) filled with commercially produced standard gases (supplied by AGA). Headspace samples (approx. 1 ml) were taken via needle and measured sequentially every 5 hours. The samples were incubated under oxic conditions for approx. 40 hours and subsequently incubated under anaerobic conditions for the remainder of the incubation (approx. 200 hours total). In order to create anoxic conditions, sampling vials were flushed and evacuated three times with high purity helium (He) gas, and over pressure was released from the vials before GC analysis.

### Calculation of C mineralization and denitrification rates

Oxic respiration (i.e. oxic C mineralization) was calculated using the mean production rates of CO_2_ (μmol/h per vial) within the first 40 hours when oxygen was present. Denitrification rates and anoxic C mineralization rates were calculated using the mean production rates of NO+N_2_O+N_2_ (μmol N/h per vial) and CO_2_ (μmol/h per vial) respectively, within the first 40 hours following removal of O_2_ by replacement of the headspace with helium.

### Calculation of N_2_O production index and (N_2_O/N_2_O+N_2_) ratio

Characterization of N_2_O emissions from each soil were done using two methods: 1) the N_2_O production index (*I*N_2_O) as described by Liu et al. [26] and Qu et al. [24] and 2) the N_2_O product ratio (N_2_O/N_2_O+N_2_) as described by Raut et al. [23]. Calculation of *I*N_2_O was done using a 5 hours interval (i.e. 0 h– 5 h, 5 h– 10 h, 10 h– 15 h, and so on), while the N_2_O/(N_2_O+N_2_) ratio only took into account a single time point (i.e. 0 h, 5 h, 15 h, and so on). All soils were compared based on a 50 h anoxic incubation period for *I*N_2_O. The N_2_O/(N_2_O+N_2_) ratio was calculated using the maximum value during the same 50 h period. Calculation of the N_2_O production index (*I*N_2_O) was done using the formula:

$$IN_{2}O=\int_{0}^{T}N_{2}O(t)dt/[\int_{0}^{T}N_{2}O(t)+\int_{0}^{T}N_{2}(t)]dt$$

where N_2_O (t) is the accumulated flux of N_2_O at any time t, N_2_ (t) is the accumulated flux of N_2_ at any time, and T is the time when a certain amount of NO_3_^—^ N g^-1^ soil is recovered as (NO_2_^-^, NO, N_2_O and N_2_)-N. Here we considered 50 h as T.

Linear regressions performed on JMP 10 (SAS Institute) were used to identify relationships between variables.

## Results

### Gas kinetics

**Fig 2:**
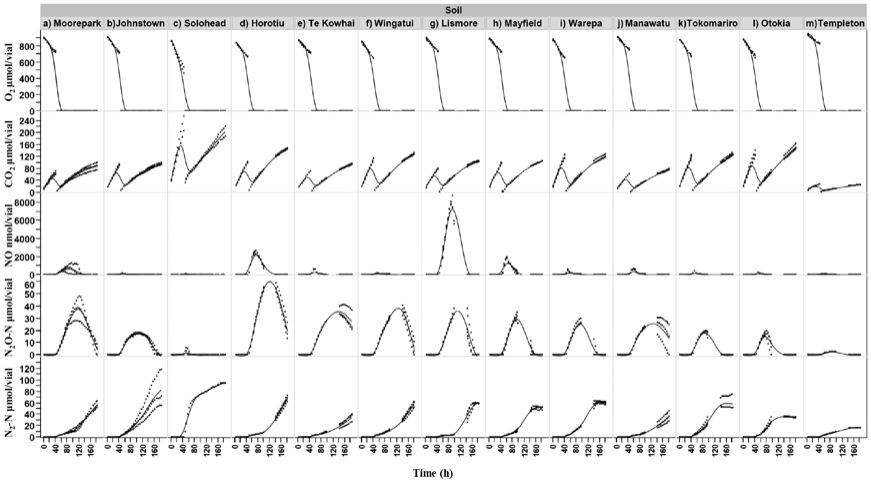
Gas kinetics profile of IR and NZ soils under oxic and anoxic conditions. O_2_, CO_2_, NO, N_2_O and N_2_ emission kinetics during incubation of 13 different temperate soils (3 Ireland (a,b,c) and 10 New Zealand (d to m)) amended with 2 mM nitrate (flooding and draining immediately before incubation). Soil samples (20 g dry weight) were incubated under oxic (first 40 hours) and subsequently anoxic conditions. Dots represent three replicate vials and smooth line is the fitted line for all data.

Soil samples incubated under oxic conditions did not produce quantifiable amounts of NO, N_2_O or N_2_ after 40 h of incubation despite active respiration as determined by consumption of O_2_ and production of CO_2_ (Fig 2). Upon removal of O_2_, immediate production of NO, N_2_O and N_2_ were detected. For all soils, NO and N_2_O were converted to N_2_, but the kinetics of the conversion varied. Accumulation of NO (mean ± SD) ranged between 100 ± 2.9 and 8390 ± 802 nmol N/vial, corresponding to Templeton and Lismore soils, respectively. While N_2_O accumulation ranged between 2.6 ± 0.5 to 56.5 ± 2.3 μmol N/vial, corresponding to Templeton and Horotiu, respectively.

### *I*N_2_O and N_2_O/(N_2_O+N_2_)

**Fig 3:**
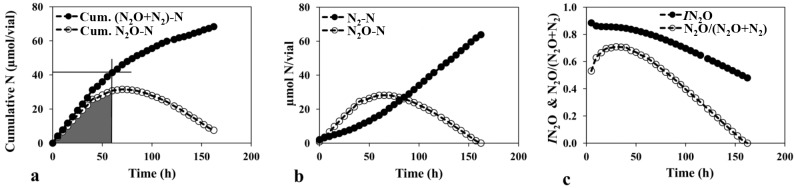
Demonstration of calculation of *I*N_2_O and N_2_O/(N_2_O+N_2_). Representative curves for a) cumulative N accumulation, b) measured N, and c) N_2_O production index (*I*N_2_O) and N_2_O/(N_2_O+N_2_) product ratio over time for one soil (Moorepark). N_2_O production indices were calculated as $IN_{2}O=\int_{0}^{T}N_{2}O(t)dt/[\int_{0}^{T}N_{2}O(t)+\int_{0}^{T}N_{2}(t)]dt$. Curves represent a single flask result. Each flask contained 20 g (dry weight) soil incubated in a 120 ml serum vial under anoxic conditions. Results for all other soils can be found in S1 Fig.

The N_2_O production index (*I*N_2_O) and the N_2_O/(N_2_O+N_2_) product ratio were calculated based on the kinetics observed during anoxic incubation (Fig 3). Soil samples displayed higher *I*N_2_O (approx. 10%) than N_2_O/(N_2_O+N_2_) with a mean value of 0.77 ± 0.27 and 0.67 ± 0.20 respectively (S2 Fig). The Solohead soil had both the lowest N_2_O production index (*I*N_2_O = 0.02) and N_2_O/(N_2_O+N_2_) product ratio (0.23), while the Lismore soil had the highest (*I*N_2_O = 1 and N_2_O/(N_2_O+N_2_) = 0.89). Values for *I*N_2_O and N_2_O/(N_2_O+N_2_) were from all soils positively associated (*r*^*2*^ = 0.84 *p*<0.001).

### Soil pH and N_2_O emissions

**Fig 4:**
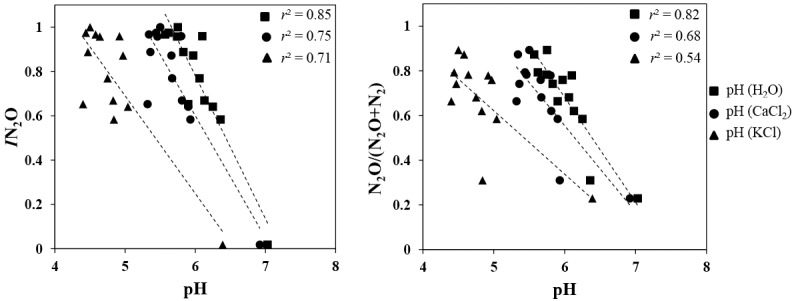
Relationship between pH and N_2_O emissions. Effect of method (extractant type) for determining soil pH on association with (a) N_2_O production index (*I*N_2_O) and (b) N_2_O/(N_2_O+N_2_) ratio. Calculation of both index and ratio was based on N_2_O emission within the curve (see S1 Fig for each sample) at 50 h under anoxic incubation. Soil pH was measured using three different extractants: i) DI water, ii) 0.01 M CaCl_2_, and iii) 2M KCl. Dotted lines represent regression lines. Points represent the mean triplicate flask results.

Soil pH values were moderately acidic to neutral across all soils (S2 Table). The pH measurements in the water-based method resulted in a wider range of values (5.57–7.03), while values for the KCl based method resulted in pH values clustered within the acidic range (4.40–6.39). The influence of soil pH on N_2_O emissions was examined by comparing pH values obtained, with each of the different pH extraction methods, with the N_2_O/(N_2_O+N_2_) ratio and the *I*N_2_O for all soils (Fig 4). Soil pH explained a significant proportion of the variation in relationship to *I*N_2_O regardless of method used to determine pH (*r*^*2*^ = 0.85 in DI H_2_O; *r*^*2*^ = 0.75 in CaCl_2_; *r*^*2*^ = 0.71 in KCl; *p*<0.05 all cases). Strong relationships (*p*<0.05) were also observed between pH and the N_2_O/(N_2_O+N_2_) product ratios regardless of soil pH extraction method (*r*^*2*^ = 0.82 in DI H_2_O; *r*^*2*^ = 0.68 in CaCl_2_; *r*^*2*^ = 0.54 in KCl). Among the soil samples, one (Solohead) displayed very low N_2_O emissions resulting in an outlier (Fig 4). To assess its impact, it was removed, and the *I*N_2_O and N_2_O/(N_2_O+N_2_) ratio were recalculated and related to pH. Only the DI water-based pH measurement was significantly associated, but the resulting *r*^*2*^ was lower (In case of *I*N_2_O and pH: *r*^2^ = 0.62 *p* = 0.0025 in DI H_2_O; *r*^*2*^ = 0.29 *p* = 0.07 in CaCl_2_; *r*^*2*^ = 0.13 *p* = 0.24 in KCl, and in case of N_2_O/(N_2_O+N_2_) product ratios and pH: *r*^*2*^ = 0.69 *p* = 0.0009 in DI H_2_O; *r*^*2*^ = 0.43 *p* = 0.019 in CaCl_2_; *r*^*2*^ = 0.17 *p* = 0.178 in KCl).

### Links between denitrification and C mineralization

**Fig 5:**
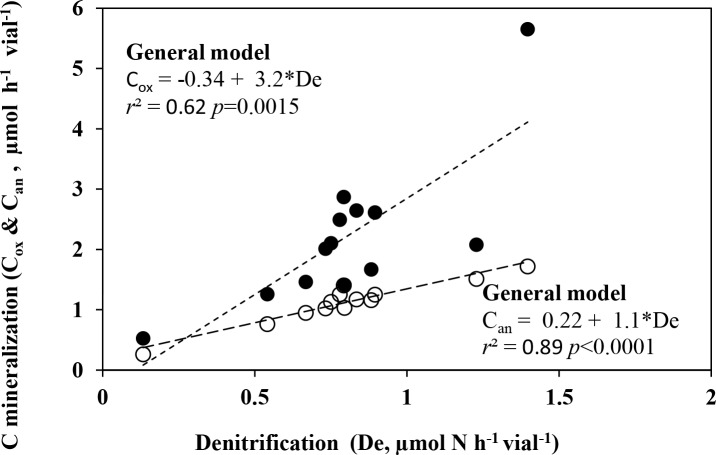
Links between denitrification and C mineralization. Relationship between C mineralization rate during both oxic [closed circles] and anoxic phase [open circles] and denitrification (De; i.e. production rates of NO+N_2_O+N_2_). Each point represents mean of triplicates. Linear regression function is shown for both oxic (C_ox_) and anoxic (C_an_) C mineralization.

The rate of soil denitrification under anoxic condition (NO+N_2_O+N_2_ μmol N/h/vial) was significantly linked to the rate of C-mineralization (CO_2_ μmol/h/vial) under both oxic (*r*^2^ = 0.62, *p* = 0.0015 and anoxic (*r*^2^ = 0.89, *p*<0.0001) conditions (Fig 5).

## Discussion

It is known that soil pH plays a strong role in regulating the loss of N gases [27]. One problem with understanding the pH effect on N_2_O emissions is consolidating the many studies done to date, and their sometimes-conflicting observations [28]. Here we tested soil pH using the three most commonly used extractants and found that soil pH measurements vary across all three extractants (approx. 1–2 units within pH range) (Fig 4 and S2 Table). This is likely due to the differences in protons (H^+^) and hydronium ions (OH^-^) attracted to exchange sites for each buffer, which causes an electrical potential to develop. Although the different pH extractants yield different soil pH values, the relative ranking of the soils from highest to lowest pH was entirely conserved across all extractants. Thus absolute values of soil pH across studies will be hard to compare but their relative placement within a gradient (higher vs. lower pH) can be used to compare results across independent studies. Evidence in the literature supports the claim for reduced N_2_O reduction and denitrification in low pH systems [23,29], leading to the N_2_O production index being strongly associated with pH [24,26]. The underlying mechanisms involved in the pH control over N_2_O emission have begun to be unravelled in part by the use of model organisms, including *Paracoccus denitrificans*. Recent work demonstrated that the relative activity of the N_2_O reductase enzyme decreased with lowering of the pH. This decrease in activity was associated with a post-transcriptional effect wherein the assembly of the N_2_OR enzyme was inhibited by low pH [19]. However, further work showed that when N_2_OR was expressed at pH 7.0, it remained functional over the entire pH range tested (5.7 to 7.6), suggesting that the role of pH is specific to the folding of the protein upon expression [20]. It is important to understand that although pH in this scenario plays a role as a proximal regulator, it can also play a role as a distal regulator as well by controlling community composition [30] making interpretation complicated.

Independent of the methods used to measure soil pH or the mechanism controlling the pattern, we observed that the *I*N_2_O was higher than the N_2_O/(N_2_O+N_2_) ratio in each soil sample. This is likely due to the fact that the *I*N_2_O takes into account a time period (the emission occurring between two given time points), as opposed to single time points as used in the N_2_O/(N_2_O+N_2_) ratio calculations. As seen in the kinetic profiles (Fig 3), the shape of the curve is not always similar and although heights (i.e. maximum values) might be similar, a gentler slope (i.e. slower but more prolonged rates) can lead to an extended period of emissions not accounted for by height alone. The fact that *I*N_2_O and N_2_O/(N_2_O+N_2_) were strongly associated (*r*^*2*^ = 0.84) suggests that both parameters can be used as a measure of the soils’ contrasting propensities to emit N_2_O. As the N_2_O production index (*I*N_2_O) is calculated using at least two time points and the area under the curve, it is possibly the best predictor of the propensity of the soils to emit N_2_O, as dependent on the ability of the denitrifying community to express N_2_O reductase. It cannot be taken as a direct predictor of N_2_O emission to the atmosphere under field conditions, primarily because the fraction of denitrification products lost to the atmosphere as N_2_O depends on soil moisture content; high soil moisture content retards N_2_O diffusion and hence increases the fraction of N_2_O reduced to N_2_.

Aside from pH, O_2_ is also a known ‘master’ regulator of denitrification. Soil samples in this study were incubated in two phases (oxic and anoxic). During the oxic phase, microbial respiration was active as determined by monitoring of the CO_2_ produced but there were no emissions of NO, N_2_O or N_2_. However, upon removal of O_2_, emissions of NO, N_2_O and N_2_ were observed in all soil samples independent of pH. This confirms prior work [23,24,26] indicating that in the hierarchy of regulators of denitrification, O_2_ serves as a primary control with pH serving a secondary role, not in controlling the rate of denitrification but the kinetics of the product ratio. Both measurements of emission potential (*I*N_2_O and N_2_O/(N_2_O+N_2_)) are strongly related to soil pH (*r*^*2*^ = 0.53 to 0.85) transient accumulation of N_2_O.

In both oxic and anoxic conditions, the C mineralization rates (CO_2_ production) for all soils provide an indirect indication of denitrification rates, and serve as a good proxy for predicting N cycling activity (Fig 5). Oxic respiration rates (or C mineralization) were 3.2 times higher than the rates of denitrification, likely due to the larger pool of organisms capable of carrying out this general process. When the anoxic C mineralization rate was compared to the rate of denitrification, a strong relationship (*r*^*2*^ = 0.89) was observed, suggesting that denitrification was the dominant pathway for energy generation and responsible for respiration from the selected soils under the experimental conditions. This is expected given the conditions used in this study favour denitrification, and its intermediates represent the most energetically advantageous alternative electron acceptor. However, we observed that C mineralization rates under anoxic conditions were 10% higher than denitrification rates, which may be due to fermentation process and/or the presence of other alternative electron acceptors in soils (e.g. Fe^2+^, Mn^2+^, SO_4_^2-^, etc.).

Although measures like C mineralization and denitrification rates, *I*N_2_O and N_2_O/(N_2_O+N_2_) allow us to assess the impact of potential regulators, as well as providing easy comparison to prior work, they do not convey all the differences observed. By using a continuous monitoring system we observed that the gas emission profile (kinetics) (i.e. the production and consumption of the gas intermediates in denitrification) of pasture soils varied greatly across all soils. Some soils (e.g. Lismore, Horotiu, Mayfield and Moorepark) were more prone to producing NO compared to others, but the profiles generated could not be summed based on a single gas. The data generated from these 13 soils suggests that our inability to accurately predict emissions is in part due to the uniqueness of each soil, which is reflected here in their unique gas profiles. Soils such as the Solohead soil would likely generate results that are difficult to interpret based on single time point measurements due to its kinetic profile (extremely fast rates of almost all measured variables). Despite these difficulties, certain conclusions can be made. Soil pH is one of the most important soil factors affecting the denitrification products (i.e. N_2_O or N_2_). Here we showed that differences in extractants for measuring pH could account for discrepancies in observations across prior studies. However, a consistent trend of increased N_2_O emissions with lowering pH was observed independent of pH extractants. Further, two approaches for representing emissions (*I*N_2_O than N_2_O/(N_2_O+N_2_)) were examined and shown to be positively associated, providing alternatives for reporting emissions. Finally, as denitrification rate is closely related to soil C mineralization, therefore C mineralization could be used as an indirect tool for predicting the denitrification rate of NO_3_^-^ amended pasture soils.

## Supporting Information

S1 Fig Emission profile of N_2_O production index (*I*N_2_O) and N_2_O/(N_2_O+N_2_) product ratio over time. N_2_O (μmol N/vial) and N_2_ (μmol N/vial) emissions from the anoxic incubation of soils over time (1a-13a), and N_2_O production index (*I*N_2_O) and N_2_O/(N_2_O+N_2_) product ratio of denitrification over time (1b-13b). Soils were collected from Ireland (1: Moorepark, 2: Johnstown, 3: Solohead) and New Zealand (4: Warepa, 5: Otokia, 6: Wingatui, 7: Tokomairiro, 8: Mayfield, 9: Lismore, 10: Templeton, 11: Manawatu, 12: Horotiu and 13: Te Kowhai). Values represent the mean and standard error of triplicate flask results.
(PDF)

S2 Fig Maximum N_2_O production index (*I*N_2_O) and N_2_O/(N_2_O+N_2_) product ratio values observed in all soils. Values represent the mean and standard error of triplicate flask results.
(PDF)

S1 Table Physical and chemical characteristics of soil samples. (XLSX)

S2 Table Soil pH measurements. Soil pH values as determined using three different extraction methods (DI water, 0.01 M CaCl_2_ and 2M KCl).
(PDF)
