# Supplementary material for: High-Resolution Denitrification Kinetics in Pasture Soils Link N2O Emissions to pH, and Denitrification to C Mineralization
Source: PLoS One. 2016 Mar 18;11(3):e0151713. doi: 10.1371/journal.pone.0151713 (PMC4798686; doi:10.1371/journal.pone.0151713)
Supplement: S1 Fig — N2O (μmol N/vial) and N2 (μmol N/vial) emissions from the anoxic incubation of soils over time (1a-13a), and N2O production index (IN2O) and N2O/(N2O+N2) product ratio of denitrification over time (1b-13b). Soils were collected from Ireland (1: Moorepark, 2: Johnstown, 3: Solohead) and New Zealand (4: Warepa, 5: Otokia, 6: Wingatui, 7: Tokomairiro, 8: Mayfield, 9: Lismore, 10: Templeton, 11: Manawatu, 12: Horotiu and 13: Te Kowhai). Values represent the mean and standard error of triplicate flask results. (PDF) [file pone.0151713.s001.pdf]

S1 Fig. Emission profile of N_2O production index (IN_2O) and $N_2O/(N_2O+N_2)$ product ratio over time. N_2O (µmol N/vial) and N_2 (µmol N/vial) emissions from the anoxic incubation of soils over time (1a-13a), and N_2O production index (IN_2O) and $N_2O/(N_2O+N_2)$ product ratio of denitrification over time (1b-13b). Soils were collected from Ireland (1: Moorepark, 2: Johnstown, 3: Solohead) and New Zealand (4: Warepa, 5: Otokia, 6: Wingatui, 7: Tokomairiro, 8: Mayfield, 9: Lismore, 10: Templeton, 11: Manawatu, 12: Horotiu and 13: Te Kowhai). Values represent the mean and standard error of triplicate flask results.

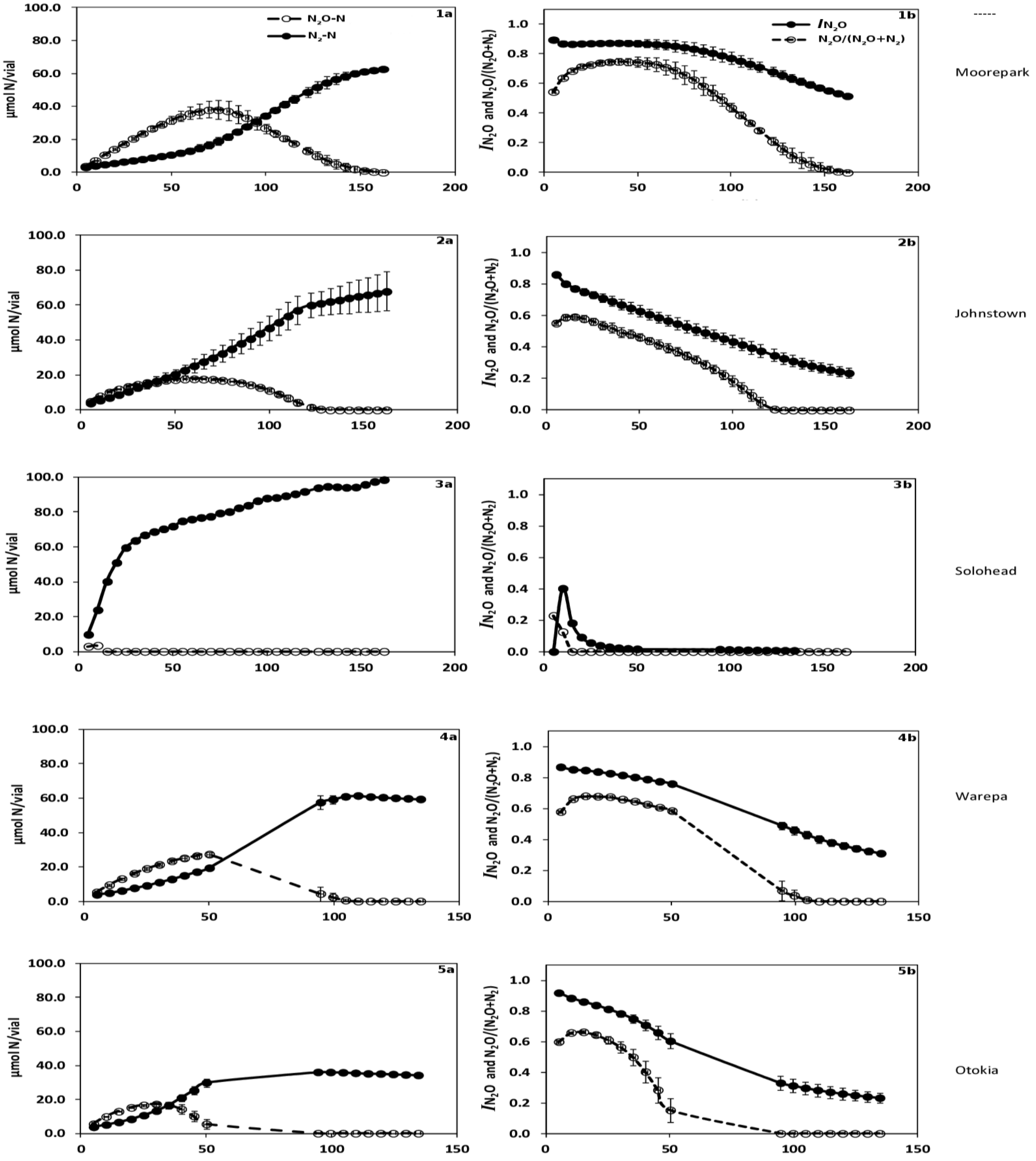

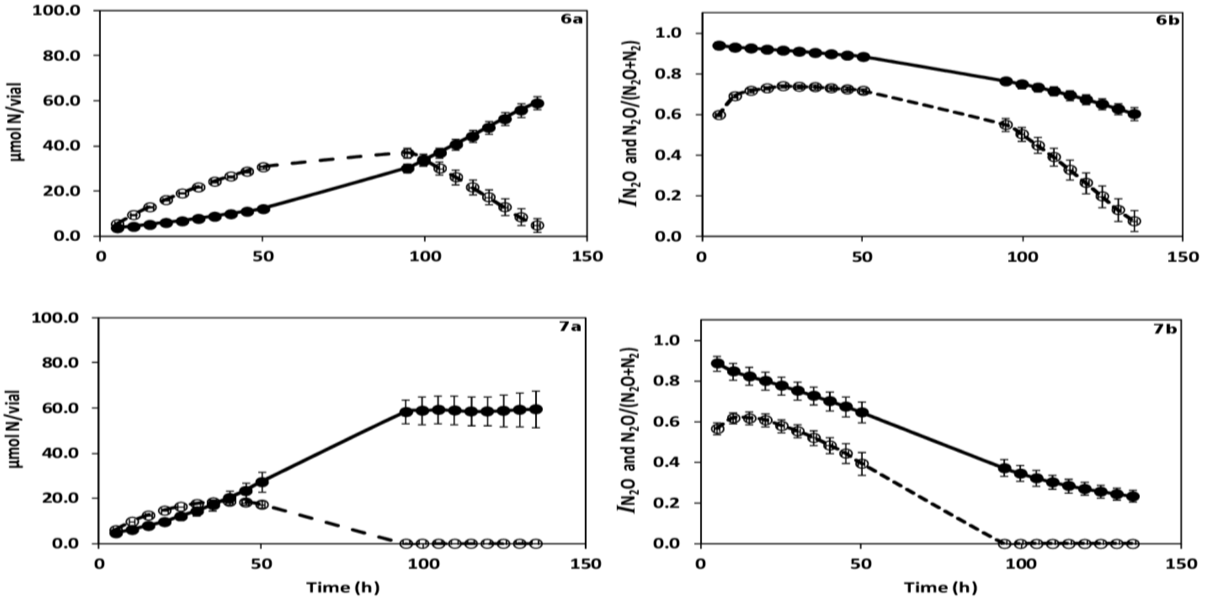

Wingatui

Tokomariro

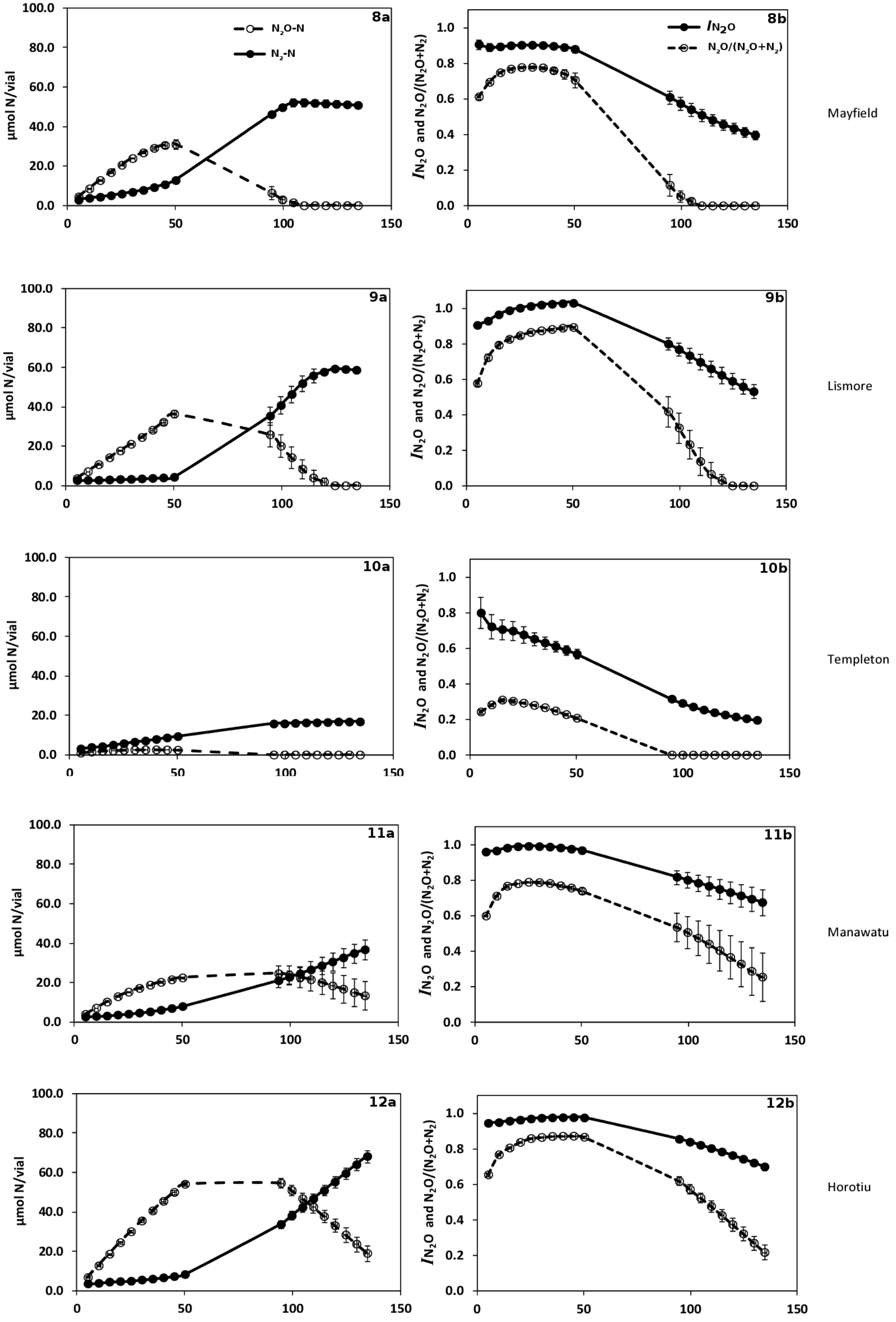
8a
9a
10a
11a
12a
8b
9b
10b
11b
12b
N2O-N
N2-N
µmol N/vial
I N2O
N2O/(N2O+N2)
I N2O and N2O/(N2O+N2)
Mayfield
Lismore
Templeton
Manawatu
Horotiu

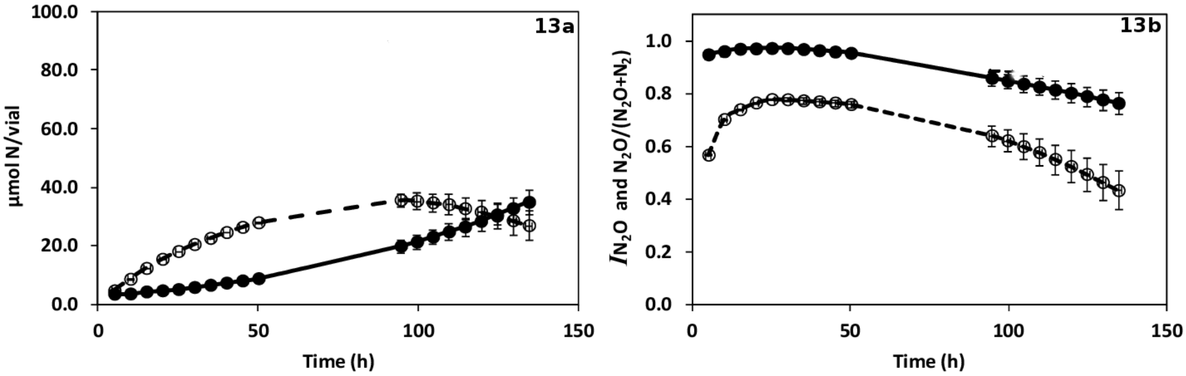

Te Kowhai
