# Supplementary material for: High-Resolution Denitrification Kinetics in Pasture Soils Link N2O Emissions to pH, and Denitrification to C Mineralization
Source: PLoS One. 2016 Mar 18;11(3):e0151713. doi: 10.1371/journal.pone.0151713 (PMC4798686; doi:10.1371/journal.pone.0151713)

S2 Fig. Maximum N_2O production index (IN_2O) and $N_2O/(N_2O+N_2)$ product ratio values observed in all soils. Values represent the mean and standard error of triplicate flask results.

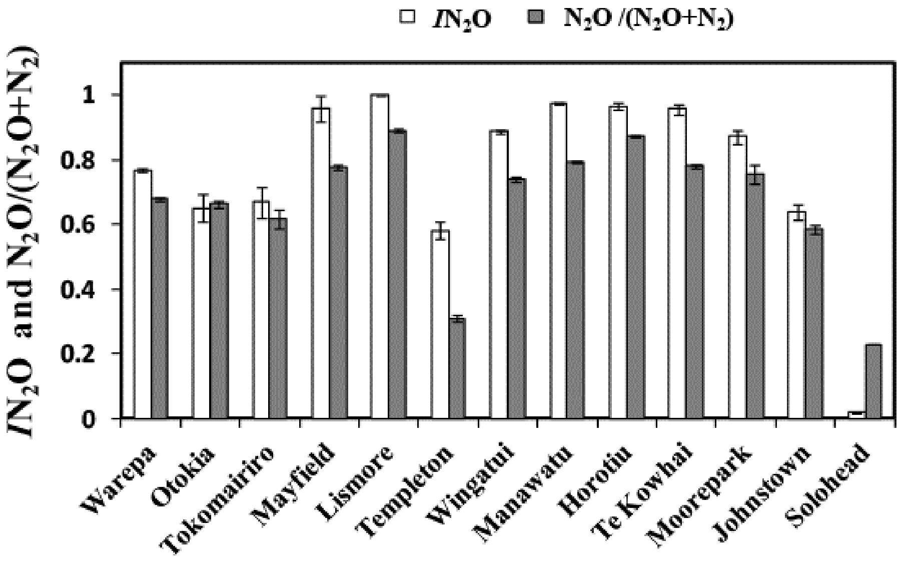

Supplement: S2 Fig — Values represent the mean and standard error of triplicate flask results. (PDF) [file pone.0151713.s002.pdf]
