# Supplementary material for: High-Resolution Denitrification Kinetics in Pasture Soils Link N2O Emissions to pH, and Denitrification to C Mineralization
Source: PLoS One. 2016 Mar 18;11(3):e0151713. doi: 10.1371/journal.pone.0151713 (PMC4798686; doi:10.1371/journal.pone.0151713)
Supplement: S2 Table — Soil pH values as determined using three different extraction methods (DI water, 0.01 M CaCl2 and 2M KCl). (PDF) [file pone.0151713.s004.pdf]

S2 Table. Soil pH measurements. Soil pH values as determined using three different extraction methods (DI water, 0.01 M $CaCl_2$ and 2M KCl).

**Soil**	**pH (H_2O)**	**pH ($CaCl_2$)**	**pH (KCl)**
Warepa	6.06	5.67	4.75
Otokia	5.90	5.32	4.40
Tokomairiro	6.13	5.81	4.83
Mayfield	6.10	5.80	4.92
Lismore	5.75	5.50	4.50
Templeton	6.36	5.93	4.84
Wingatui	5.83	5.36	4.47
Manawatu	5.62	5.44	4.44
Horotiu	5.57	5.34	4.58
Te Kowhai	5.74	5.46	4.64
Moorepark	5.97	5.66	4.97
Johnstown	6.25	5.90	5.04
Solohead	7.03	6.92	6.39
